# Role of Selenium in Viral Infections with a Major Focus on SARS-CoV-2

**DOI:** 10.3390/ijms23010280

**Published:** 2021-12-28

**Authors:** Sabrina Sales Martinez, Yongjun Huang, Leonardo Acuna, Eduardo Laverde, David Trujillo, Manuel A. Barbieri, Javier Tamargo, Adriana Campa, Marianna K. Baum

**Affiliations:** 1 Robert Stempel College of Public Health and Social Work, Florida International University, Miami, FL 33199, USA; saless@fiu.edu (S.S.M.); hyongjun@fiu.edu (Y.H.); jtamargo@fiu.edu (J.T.); campaa@fiu.edu (A.C.); 2 College of Arts, Sciences & Education, Florida International University, Miami, FL 33199, USA; lacuna@fiu.edu (L.A.); eelaverd@fiu.edu (E.L.); datrujil@fiu.edu (D.T.); barbieri@fiu.edu (M.A.B.)

**Keywords:** selenium, selenoproteins, virus, viral, infection, reactive oxygen species, antioxidant, HIV, HCV, HBV, coxsackie virus, influenza, glutathione peroxidase, thioredoxin reductase

## Abstract

Viral infections have afflicted human health and despite great advancements in scientific knowledge and technologies, continue to affect our society today. The current coronavirus (COVID-19) pandemic has put a spotlight on the need to review the evidence on the impact of nutritional strategies to maintain a healthy immune system, particularly in instances where there are limited therapeutic treatments. Selenium, an essential trace element in humans, has a long history of lowering the occurrence and severity of viral infections. Much of the benefits derived from selenium are due to its incorporation into selenocysteine, an important component of proteins known as selenoproteins. Viral infections are associated with an increase in reactive oxygen species and may result in oxidative stress. Studies suggest that selenium deficiency alters immune response and viral infection by increasing oxidative stress and the rate of mutations in the viral genome, leading to an increase in pathogenicity and damage to the host. This review examines viral infections, including the novel SARS-CoV-2, in the context of selenium, in order to inform potential nutritional strategies to maintain a healthy immune system.

## 1. Introduction

Viral infections have afflicted human health despite great advancements in scientific knowledge and technologies [1,2,3]. Most recently, the novel severe acute respiratory syndrome coronavirus 2 (SARS-CoV-2) has infected over 200 million individuals during 2019–August 2021 and has led to over 4.4 million deaths globally [4]. Selenium (Se), an essential trace element in humans, has a long history of lowering the occurrence and severity of viral infections [5,6,7,8,9]. Se deficiency impacts immune function [10], viral expression [8], selenoprotein expression [11], and alters antioxidant response [12], allowing for greater susceptibility to severe viral and bacterial infections [13]. Supplementing the diet with Se has demonstrated positive effects on enhancing immunity against viral attacks [5]. Much of the benefits derived from Se are due to its incorporation into selenocysteine, an important component of the antioxidant defense systems, including the regulation of glutathione peroxidase (GPXs) and thioredoxin reductase (TXNRD) activities [14]. Low levels of Se can lead to more severe forms of viral infections and adequate selenium levels may provide a protective effect toward the host response by affecting both immune response and oxidative stress [13,15]. Severe pathology in Se deficiency is evidenced by more frequent and graver symptoms, higher viral loads, declining levels of antioxidant enzymes such as GPX, and mutations to the viral genome. Studies conducted by Beck et al. described in this review, demonstrate that Se-deficiency is capable of increasing the virulence of a benign coxsackie virus through viral mutations and these mutations have led to a reduction in GPX activity, therefore, resulting in oxidative stress [13,15]. 

The current coronavirus (COVID-19) infection pandemic has put a spotlight on the need to review the evidence on the impact of nutritional strategies to maintain a healthy immune system, as there are limited therapeutic treatments. Therefore, this review principally focuses on Se, in the context of viral infections, including the novel SARS-CoV-2. A review of the most common selenoproteins and their functions will be followed by the evidence on the role and impact of Se on the human host’s ability to battle viral infections. 

## 2. Selenoproteins and Functions

Selenoproteins are proteins that have incorporated the 21st amino acid in the genetic code, selenocysteine (Sec) into their polypeptide chain. Selenocysteine is a true proteinogenic amino acid in that it has its own unique codon (UGA), Sec insertion sequence (SECIS), Sec-specific elongation factor (eEFsec), transfer RNA (tRNASec), and is co-translationally inserted [16]. The biological functions of Se are mostly exerted through selenoprotein domains that contain Sec residues [17,18]. Twenty-four selenoprotein genes have been characterized in mice and 25 in humans [19,20]. Some of these selenoproteins demonstrated their essential roles in developmental processes and in disease pathogenesis [21,22]. Selenoproteins have been classified based on their known or suspected cellular functions; for example GPX 1–4 for antioxidation, TXNRD 1–3, methionine sulfoxide reductase B (MSRB)1, selenoproteins (SELENO) H, M, and W for redox regulation, iodothyronine deiodinase (DIO) 1–3 for thyroid hormone metabolism, SELENOP for selenium transport and storage, selenophosphate synthetase (SEPHS) 2 for the synthesis of selenophosphate, SELENOK and T for calcium metabolism, SELENON protein involved in myogenesis, SELENOF, I and S for protein folding, and SELENOO protein with AMPylation activity [21,22]. 

Only 2 of the 25 selenoproteins identified are extracellular, selenoprotein P (SELENOP), and extracellular glutathione peroxidase (GPX3) [23]. SELENOP is noteworthy in that it carries out the crucial role of distributing Se in plasma from the liver where dietary selenium is metabolized [24,25]. and contains up to 9 Sec residues [23]. SELENOP then binds to apolipoprotein E receptor-2 (apoER2) receptors on various tissues including the brain and testis or lipoprotein receptor megalin (Lrp2) for endocytosis in the kidneys for systemic distribution [18,26]. Different isoforms of SELENOP confer specificity to the various receptors [26]. Once endocytosed, Se can be used for the formation of other selenoproteins. 

Among the more well-studied selenoproteins are those involved in maintaining homeostatic redox states, namely GPXs. There are 5 isoforms of GPXs that contain selenocysteine residues and they each occupy distinct regions of the cell. Each GPX isoform catalyzes the reduction of hydrogen peroxides using glutathione (GSH) as a cofactor, and in doing so, maintains cellular homeostasis. In this capacity, GPXs play a vital role not only in the prevention of oxidative stress but also in regulating redox signaling that can have broader effects on cell proliferation, apoptosis, and cytokine expression [27]. This important role of GPX and dietary Se is highlighted by the work of Beck et al., described later in this review, which demonstrated that Se-deficient mice were susceptible to a myocarditic strain of coxsackievirus whereas Se-adequate mice were unperturbed [10,28]. It was hypothesized that diminished activity of GPX was responsible for viral mutations in the Se-deficient mice and the production of more pathogenic virions [10,28]. 

Thioredoxin reductases (TXNRDs) are a family of selenoproteins, whose main function is to reduce thioredoxins but has broad specificity allowing it to reduce other endogenous and exogenous substrates [18,29]. The reduction of TXNRD’s is accomplished by electrons from nicotinamide adenine dinucleotide phosphate (NADPH), which are transferred to the active site of TXNRDs via flavin adenine dinucleotide (FAD), a redox-active coenzyme [18,29]. Thioredoxins themselves reduce a number of small proteins including transcription factors such as nuclear factor kappa beta (NF-κβ), p53, redox factor 1 (REF-1), apurinic/apyrimidinic endonucleases 1 (AP-1), and phosphatase and tensin homologue deleted on chromosome ten (PTEN) thereby controlling the expression of various genes involved in cell growth, proliferation and inflammation [30]. 

Methionine sulfoxide reductase (MSR) is yet another selenoprotein with enzymatic activity that combats intercellular oxidative damage [18,31,32,33]. Specifically, MSR reduces the oxidized sulfur of methionine sulfoxide to produce the amino acid methionine [18,31,32,33]. Methionine sulfoxide alters protein function, may cause misfolding and dysregulates key cellular processes [33]. Lee et al. [32] demonstrated that MSRB1 is involved in cytokine regulation in macrophages by promoting the expression of anti-inflammatory cytokines IL-10 and IL-1RA. Coincidently, MSRB1 is the only methionine sulfoxide reductase that is a selenoprotein [32].

Unlike the aforementioned selenoproteins, SELENOK does not participate directly in redox reactions [34]. Instead SELENOK, a disordered endoplasmic reticulum transmembrane protein is reliant on partner proteins to form complexes and execute various functions [34]. One of the most well-established roles of SELENOK is in the palmitoylation of various substrates when complexed with the acyltransferase DHHC6 [34]. One target of the SELENOK/DHHC6 complex is inositol 1,4,5-trisphosphate receptor, an endoplasmic reticulum (ER) calcium channel protein that is stabilized once acylated [34]. SELENOK, therefore, plays a role in maintaining calcium efflux that is necessary for cell survival and immune cell responses [34].

## 3. Viral Infections, Reactive Oxygen Species, and Selenium

Viral infections are associated with an increase in reactive oxygen species (ROS), which are known to have both favorable and unfavorable effects on the host’s cells and are important for the viral processes to maintain their infectious cycle [35,36]. ROS are a collection of molecules originating from molecular oxygen produced through redox reactions. Radical, having one free electron, and non-radical ROS may be formed by the partial reduction of oxygen [37,38]. Within the host cells, a balance between ROS production and ROS scavengers exists, where viral infections may create an unbalanced situation that develops into oxidative stress [36]. ROS scavengers and antioxidant systems that help to maintain redox homeostasis include catalase (CAT), superoxide dismutases (SODs), GPXs, TXNRDs, peroxidredoxin (PRDXs), and GSH. If oxidative stress remains unchecked, ROS may damage cellular proteins, lipids, and nucleic acids leading to adverse health effects and increasing the risk for several diseases [38,39]. 

Selenium plays a major role in redox regulation via its incorporation in the form of selenocysteine, into a family of proteins called selenoproteins [6]. Among these proteins, GPXs and TXNRDs play a critical role as antioxidants and confer protection against free radicals released by the immune response as a result of viral infection [8]. TXNRD defense involves the regulation of nuclear factor erythroid 2–related factor 2 (Nrf2) activation, which protects the cell against oxidative stress and inflammation [40], while GPX antioxidant defense involves the reduction of various hydroperoxides and oxidized antioxidants by catalyzing the conversion of GSH to glutathione disulfide [9]. Membrane integrity is also maintained through GPXs [41]. Studies have shown that inadequate Se intake affects GPX and TXNRD levels compromising cell-mediated immunity and humoral immunity linked to an increased inflammatory response by the production of ROS and redox control processes [40,42]. ROS production increases the expression of proinflammatory cytokines such as tumor necrosis factor-alpha (TNF-α) and interleukin (IL)-6, through the upregulation of NF-κβ activities [42]. Selenium acts as a crucial antioxidant through the modulation of ROS production by inflammatory signaling inhibiting the activation of NF-κβ cascade and suppressing the production of TNF-α and IL-6 [43]. Low Se levels decrease antioxidant activity thus decreasing free radical neutralization [44]. These studies suggest that Se deficiency alters immune response and viral infection by increasing oxidative stress and the rate of mutations in the viral genome, producing an increase in pathogenicity and damage to the host, as reported on influenza and coxsackie viruses [6]. 

## 4. Viral Infections and Selenium

### 4.1. Coxsackie Virus

Several decades of research have provided sufficient evidence to demonstrate a relationship between Se deficiency and Keshan disease, a grave cardiomyopathy. This cardiomyopathy is believed to be caused by infection with Coxsackie B virus, a nonenveloped single-stranded RNA virus pertaining to the *Picornaviridae* family, and exclusively found within China [10,45]. It was later discovered in the 1970s and 1980s that much of the Se levels in the soil, water, food, and human circulating fluids in areas affected by Keshan disease were deficient compared to other neighboring Chinese providences [46]. Sodium selenite was provided to the population and a prospective study showed that it prevented Keshan disease. Keshan disease was eradicated from endemic areas after the government enacted a Se supplementation policy, therefore, demonstrating that Keshan disease occurred due to two factors, infection with Coxsackie B virus and Se deficiency [47,48,49]. 

Animal studies conducted by Beck et al. confirmed the relationship with Se in mice by infection with a non-cardio-virulent strain of Coxsackie B virus (CVB3/0) and a myocarditic strain (CVB3/20). Heart damage was only observed in the mice fed a Se-deficient diet compared to mice fed a Se-sufficient diet for 4 weeks, and the typical human pathology was also observed [28,50]. These studies illustrated that Se deficiency caused a virus that was non-virulent to contribute towards the development of myocarditis in the host, and also increased its pathogenicity as the cardiovirulent strain under Se deficiency produced greater symptoms [28,51]. 

Additional observations by Beck and colleagues showed higher viral loads in the Se-deficient mice infected with both CVB3/0 and CVB3/20. The Se-deficient mice were found to have reduced T-cell expansion and diminished mRNA levels of cytokines compared to Se-adequate mice [15]. Subsequent studies led to the finding that Se deficiency was responsible for a change in the genotype of the benign coxsackie virus CVB3/0 that caused it to become virulent. Specifically, six nucleotides were modified that mimicked other virulent strains of CVB3 viruses. Due to these mutations, the virus now had the possibility to become pathogenic even in a Se-adequate host [45]. It was then hypothesized that a reduction in GPX activity was responsible for the viral mutations. Therefore, subsequent studies were conducted to demonstrate the protective effect of GPX1 in developing heart damage when infected with a benign strain of Coxsackie B virus (CVB3/0) [52]. Mice with a disrupted *gpx1* gene infected with CVB3/0 compared with wild type mice with an intact *gpx1* gene experienced myocarditis, and sequencing of the viruses from the mice with disrupted *gpx1* gene showed seven nucleotide changes in the Coxsackie virus. Interestingly, six of the seven nucleotide changes in the genome of the virus from the mice with disrupted *gpx1* genes matched the changes found in the Se-deficient mice previously [52]. These classic experiments exhibit how nutritional status as it pertains to Se, and its ability to protect antioxidant systems and immunity may impact the potential evolution of viruses to become more virulent. 

### 4.2. Influenza

Influenza viruses, known to cause the flu, are enveloped, single-stranded RNA viruses within the *Orthomyxoviridae* family. Selenium deficiency has been associated with poor selenoprotein expression [11] and altered antioxidant response in viral influenza A infection [12]. The elegant in vitro [13] and animal experiments conducted by Dr. Beck et al. [28,45,46,50,52,53] were the first to demonstrate the detrimental effects of Se deficiency in influenza A virulence, which occurred due to changes in the viral genome [54]. Se deficiency in mice infected with a highly virulent Influenza A strain (Influenza A/PR/8/34), however, had higher levels of IL-2 expression followed by a higher level of IL-4 expression in the lung, and higher survival compared to Se-adequate mice. These studies demonstrated the essential role of Se in mounting an immune response to influenza A, by changing its virulence and altering the host’s immune response [55]. 

These in vitro and animal studies suggested that in vivo Se supplementation might have a beneficial effect in humans, especially in the elderly, as the immune response is compromised by age. To test this hypothesis, Ivory et al. [56] conducted a 12-week randomized, double-blinded, placebo-controlled clinical trial in six groups of individuals with suboptimal Se status or plasma Se levels < 110 ng/mL to observe the response after the flu vaccine was provided. Four groups were given daily capsules of yeast: 20 participants were given 0 μg Se/day (placebo), 18 participants were given 50 μg Se/day, 21 participants received 100 μg Se/day, and 23 received 200 μg Se/day. Two groups were given onion-containing meals, 17 participants received < 1 μg Se/day (unenriched onions), and 18 participants received 50 μg Se/day (Se enriched onions). After 10 weeks of supplementation, all participants were administered the flu vaccine. Selenium supplementation compared to placebo had beneficial and detrimental effects on the cell immunity response to the flu vaccine that was dependent on the type of Se, and dose administered [56]. Se-yeast dose of 200 µg/day demonstrated enhanced IL-10 secretion and lower granzyme B content, a cytotoxic protease that induces apoptosis of target cells, within a cluster of differentiation 8 (CD8) cells, while 50 µg/day of Se through the enriched onion meal increased granzyme content and perforin in CD8 cells and reduced natural killer T-cells. 

The effectiveness of antiviral agents such as amantadine (AM) [57], oseltamivir (OTV) [58], β-thujaplicin (TP) [59], and ribavirin (RBV) [60] to combat viral influence has been limited by the emergence of drug-resistant viruses. Biological Se nanoparticles are increasingly used as an agent to diminish drug resistance by “decorating” the nanoparticles with antiviral drugs to increase effectiveness, such as Se@AM, Se@OTV, Se@TP, Se@RBV. Selenium nanoparticles have been found to decrease oxidative stress, induce apoptosis of infected cells, and reduce lung cell damage during influenza infection, in addition to having low toxicity and increased drug activity in murine [59] and in vitro models [60].

### 4.3. Human Immunodeficiency Virus (HIV)

It is estimated that over 37 million people globally are living with HIV [61]. HIV is an enveloped, single-stranded RNA virus and without treatment causes a collapse of the immune system. The prevalence of Se deficiency in people living with HIV (PLWH) is reported to be around 7–66% and increases as HIV disease progresses over time [62,63,64,65]. Although antiretroviral therapy (ART) has allowed HIV disease to become a chronic disease, the immune system is still not fully reconditioned [66]. The rate of Se deficiency in PLWH in Sub-Saharan Africa is greater than that in the United States (U.S.A.) and the literature shows lower Se soil content in Sub-Saharan Africa [67]. Selenium deficiency in HIV disease is associated with disease progression and mortality, regardless of ART initiation [63,68,69,70,71]. Models of simian immunodeficiency virus also corroborate the relationship between Se deficiency and disease progression [72].

The relationship between HIV disease and increased oxidative stress [73,74,75,76,77,78] was recorded early in the disease, and the development of ROS and its association with HIV disease progression was documented in the very early stages of the emergence of the disease [79,80]. Lower GSH levels were found as HIV advances to acquired immunodeficiency syndrome (AIDS) [81] and alterations in antioxidant defense systems (SOD, CAT, and GPX) have also been observed in PLWH [75,77,82]. Supplementation of 250 µg of L-selenomethionine (100 µg of Se) for one year led to increased GPX activity [83] and adequate dietary Se intake was also associated with lower oxidative stress in PLWH [84].

Studies in children and adults living with HIV have found associations with Se deficiency and adverse health outcomes including mortality. Countries with a high prevalence of HIV such as South Africa, have shown that Se intake in children is not adequate and the overall diet quality is low [85]. In studies conducted in Nigeria, children with HIV had significantly lower Se levels compared to matched HIV-non-infected children in the same region and a high rate (>70%) of Se deficiency [86,87]. In children living with HIV in the U.S.A., Se deficiency was associated with advanced immunodeficiency [88] and mortality [63]. In adult PLWH who were initiating antiretroviral therapy (ART) or were already taking ART, Se deficiency was associated with HIV disease progression and mortality [70,71]. Additionally, Se values have been found to be lower in adult PLWH than in adults without HIV [89,90], as well as in later stages of HIV [90].

Several Se supplementation trials have been conducted within the United States [91,92], Tanzania [93,94], Botswana [95], and Rwanda [96]. These trials have demonstrated that Se supplementation in the dose of 200 µg in PLWH who are ART naïve or on ART may delay HIV disease progression through maintenance of cluster of differentiation 4 (CD4) cell counts. Hurwitz et al. [92] demonstrated that supplementation with Se resulted in significantly suppressed HIV viral load along with improved CD4 cell count. Trials using Se as part of a formula in combination with other micronutrients have not been able to discern the benefits of Se from the other components. We [95] concluded that supplementation with multivitamins and Se was safe and statistically significantly reduced the risk of immune decline and morbidity. Discrepancies between supplementation studies include the ART status of the participants, the baseline CD4 cell counts, and the length of time that the participants were supplemented and followed [97]. A Cochrane review of micronutrient supplementation and HIV concluded that additional trials with single nutrients were needed to build the evidence base for adults and establish long-term benefits [98]. 

### 4.4. Hepatitis B and C Viruses (HBV and HCV)

The World Health Organization (WHO) estimates that 257 million people and 71 million people were infected with hepatitis B virus (HBV) and hepatitis C virus (HCV), respectively [99]. Both HBV and HCV can cause acute and chronic hepatitis which can develop into cirrhosis and hepatocellular carcinoma (HCC). In 2015, there were 720,000 and 470,000 deaths from hepatic cirrhosis and HCC, respectively [99]. Though both HBV and HCV are hepatotropic, HCV belongs to the Flaviviridae family, whereas HBV is a member of the Hepadnaviridae family [100]. HBV is a partially double-stranded DNA virus that uses the host RNA polymerase II machinery to produce pre-genomic RNA, which is reverse transcribed into viral DNA [101]. HCV is an enveloped, single-stranded RNA virus, which exhibits extremely high mutation rates—up to one mutation per genome per generation cycle—since proofreading activity is lacking in RNA-dependent RNA polymerases required for its replication [102]. 

Selenium status determined by GPX3 activity and the concentration of serum/plasma Se and plasma SELENOP have been reported to be influenced in HBV and HCV patients in several studies. Serum Se concentrations are statistically significantly lower in HBV/HCV infected people when compared with the control group [103,104]. Selenium level is also associated with the severity and progression of the HBV/HCV disease [103,105,106]. Increased concentrations of aspartate aminotransferase and alanine aminotransferase (ALT) were independently associated with low Se concentration in chronic HBV patients with more hepatic damage [106]. The Se concentrations in plasma and erythrocytes are significantly lower in HCV-infected people than in controls and have an inversed correlation with HCV viral load [107]. Besides this, plasma Se level is statistically lower in people with HCV-induced cirrhosis with and without HCC when compared with HCV-infected people without liver cirrhosis or HCC [108]. 

Chronic HBV and HCV infection enhances ROS production and cause elevated oxidative stress and decreased antioxidant activity in liver cells [109,110,111,112]. ROS, produced as byproducts during cellular metabolism, have been implicated in several hepatic pathologies to maintain cellular homeostasis, including cell signaling, transcription, apoptosis, and immunomodulation [113,114,115,116]. Patients suffering from HBV or HCV infection show significant depletion of GSH and GPX when compared to non-infected participants [117,118]. As part of the antioxidant defense system, Se deficiency may be enhanced by the hepatic viral-induced oxidative stress and the requirement of selenoproteins during viral replication. An in vitro study showed that HCV can inhibit the expression of gastrointestinal-GPX (GPX2), a GPX that is also expressed in the liver, resulting in an increase in viral replication [7,119]. Nonstructural protein 5A (NS5A) of HCV, which is reported to enhance oxidative stress by perturbing Ca^2+^ homeostasis [120], also induces the expression and activity of GPX1 and GPX4 [121]. Besides, the GPX homology region overlaps the highly conserved *NS4* gene in HCV, supporting that the *NS4* gene is a functional GPX module [122]. Although the causes of Se deficiency in HBV and HCV are not fully understood, it is possible that the decreased level of circulating Se is related to the requirement of Se during viral replication.

The demand for Se during HBV and HCV infection causes the systemic deficiency of Se and can be compensated by supplementation. Supplementation of Se has shown to be protective against a wide range of different sources of oxidative stress and optimal immune responses [123,124]. Primary HCC incidence was reduced by 35.1% in Se supplemented people living with HBV as compared with non-supplemented people living with HBV [125]. However, when Se supplementation was stopped, primary HCC incidence began to increase [125]. Selenium also improves the rate and level of antibody response against the HBV vaccine in insulin-dependent diabetes mellitus cases that were on an accelerated vaccination schedule instead of a routine vaccine schedule [126]. A triple antioxidant combination of Se, alpha-lipoic acid, and silymarin supplementation in three chronic HCV-infected patients demonstrated an improvement in ALT [127]. However, a 6-month trial showed that those living with HCV supplemented with vitamins C, vitamin E, and 200 μg Se per day had an increase in antioxidant status with no beneficial effect on ALT, HCV viral load, or liver damage as compared with the non-supplemented individuals living with HCV [128].

As discussed in the previous paragraph, Se deficiency has been involved in the pathogenesis of HBV and HCV infection. In turn, the deficiency of Se leads to elevated oxidative stress, pathological changes, and inflammation in the liver [129]. Histological study shows hepatic sinus expansion, lymphocyte infiltration, and stripe-like hyperplasia in the liver with Se deficiency. Liver inflammation is initiated by Se deficiency as pro-inflammatory factors and molecules, such as IL-1β, IL-6, IL-12, NF-κβ, and NF-κβ p65, were all significantly higher in the Se-deficient group [129]. Hepatic antioxidant capacity is also influenced by Se deficiency as a decrease in both mRNA expression of selenoprotein genes (GPX1 and GPX3), as determined by quantitative real-time PCR and the level of selenoproteins (GPX1, GPX4, and TXNRD1), identified by global proteomics, are observed [129,130,131]. Interestingly, an in vitro study showed that Se deficiency can result in oxidative stress and apoptosis of non-HBV-infected hepatocytes, whereas HBV-infected hepatocytes gain a survival capacity and escape from the apoptosis consequence [132].

### 4.5. Poliovirus

Poliovirus is part of the *Picornaviridae* family of RNA viruses that are non-enveloped and may infect vertebrate animals [133]. Infection generates high levels of ROS and reactive nitrogen species as well as antioxidant enzymes being downregulated within cells that have been infected [134]. The supplementation of Se has been shown to improve the response of the vaccine for the poliovirus more in patients that have less optimal immune systems based on Se status, although the impact of supplementation on patients with optimal immune systems based on Se status is unclear [135]. Furthermore, the supplementation of Se did not affect all aspects of an individual’s immune response shown in the same trial where a live poliomyelitis vaccine was given to people with low Se status. This resulted in the increase of T cell and IL-10 production but did not affect the natural killer (NK) or B cell count, still resulting in the rapid removal of poliomyelitis from the patients supplemented [136]. Selenium also did not affect the levels of CD4+ T helper (Th) 1 cells to Th2 cells or the humoral immune response [135] in a different trial where patients were given a dose of the poliovirus vaccine and took either a placebo, 50 µg or 100 µg of Se. An increase in the antibody titers within all groups that were relatively equal was shown [135]. Se supplementation prior to the polio vaccine seemed to only enhance the cellular antiviral immune response. 

### 4.6. Severe Acute Respiratory Syndrome Coronavirus 2 (SARS-CoV-2)

The novel COVID-19 is caused by SARS-CoV-2, a single-stranded RNA coronavirus. The severity of the disease has been linked to aging and comorbidities such as hypertension, diabetes, obesity, cardiovascular disease, kidney disease, cancer, and pulmonary diseases [137,138]. Most of the people who test positive for COVID-19 develop mild or no symptoms, while others develop acute respiratory distress syndrome (ARDS), heart failure, blood clots, neurological complications, and elevated inflammatory response [137,139]. SARS-CoV-2 pathology has been associated with an increased immune response, leading to a release of cytokines and chemokines, also known as cytokine storm [140], as well as increased inflammatory markers such as D-dimer and ferritin [141,142]. This hyperactive inflammatory response may also bring about severe pathology in the brain [143]. SARS-CoV-2 may directly impact the central nervous system and enter the brain through various routes [144,145,146,147]. Increased systemic inflammation promoted by SARS-CoV-2 has the potential to disturb the blood-brain barrier and co-morbidities associated with severe cases of COVID-19 may enable the attack of the brain by SARS-CoV-2 [143,148]. 

It has been noted that there is a potential and developing relationship between Se levels and COVID-19 outcomes. Proposed mechanisms by which Se may act upon the SARS-CoV-2 virus based on previous research in RNA viruses include restoration of GPX and TXNRD thus reducing oxidative stress, reduction of viral-induced cell apoptosis, provision of Se for the host’s antioxidant needs, protection of endothelial cells, and reduced blood platelet aggregation [149,150]. COVID-19 is associated with a heightened level of oxidative stress and inflammation that are implicated in the pathogenesis of pulmonary disease [151]. GSH provides protection to the epithelial barrier within the lungs, and it has been suggested that improvement of GSH levels would be a strategy that may protect against inflammation and oxidant-related damage in the lungs [151]. A study conducted by Mahmoodpoor et al. [152] supplemented sodium selenite in patients with ARDS, often associated with severe cases of COVID-19, and found that it restored the antioxidant capability of the lungs, reduced inflammation, and improved respiratory mechanics. Lower total lymphocytes and CD4+ T, CD8+ T, B, and NK cells were found in COVID-19 patients and those with severe cases compared to mild cases of COVID-19 had lower lymphocyte subsets [153]. The function and differentiation of B and T cells may be affected by Se status [154]. Deficiency of Se in mice has been associated with lower T cell proliferation, while supplementation increased T cell activity and differentiation [155]. 

Clinical data investigating Se and COVID-19 are sparse; however, some reports from China and other countries globally have surfaced. In China, where there is a wide range of soil Se levels and thus a variation of Se daily intake, a linear association has been demonstrated between reported cure rates of COVID-19 and Se hair concentration data, dating from 2011 and older [156]. The same research group in China documented higher fatality risk in cities that had selenium-deficient levels in crops and topsoil compared to cities with non-deficient selenium levels in crops and topsoil [157]. Intake of Se varies worldwide, and China is known to be one of the most Se deficient countries in the world, with a wide range of levels that differs from lowest to highest in the world. COVID-19 fatality rate varies across different regions in China, suggesting that Se status may be related to COVID-19 outcomes [156,158]. In the city of Wuhan, where the SARS-CoV-2 virus was first discovered, and in other cities such as Suizhou and Xiaogan, low Se soil status was associated with the highest COVID-19 incidence [156]. In contrast, cities such as Enshi, Yichang, and Xiangyan, where high Se intake occurs, had the lowest COVID-19 incidence [156]. In contrast, in a retrospective study completed in Wuhan, China, with hospitalized COVID-19 patients, the severity of COVID-19 was associated with higher Se levels in urine [159]. The authors hypothesize that liver abnormalities due to the severity of the disease may have impacted the excess urinary Se found in severe COVID-19 patients [159]. 

Studies conducted in other parts of the world are showing similar relationships to those completed in China. In a study conducted in South Korea on hospitalized COVID-19 patients, 42% were found to be Se deficient and as the severity of disease increased, Se plasma levels decreased [160]. These patients also experienced additional nutritional deficiencies. COVID-19 patients compared to healthy controls in India, Iran and Russia had significantly lower plasma Se levels [161,162,163]. A greater rate of low plasma Se levels (<70 ng/mL) was found in COVID-19 patients (43%) compared to controls in India (20) [161]. Lung damage, as assessed by computer tomography, was inversely associated with Se levels in Russia [163]. 

COVID-19 patients may also experience increases in oxidative stress and increases in Se-related markers and lower Se levels have been documented in these patients. Moghaddam al. [164] observed an association between markers of Se status and COVID-19 outcomes from COVID-19 patients in Germany. Serum Se and SELENOP concentrations were lower in COVID-19 patients compared to a reference European population. A comparison of patients that survived compared to those who died from COVID-19 showed that the deceased had a significantly greater deficiency of serum Se and SELENOP concentrations than those who survived. In addition, those who died had significantly lower serum Se, SELENOP levels, and GPX compared with patients who survived. A study in Belgium using a convenience sample of patients hospitalized with severe COVID-19 pneumonia observed statistically lower GSH levels and higher GPX levels compared with reference intervals among other results showing elevated markers of oxidative stress and lower antioxidant status [165]. Recently, Polonikov [166] hypothesized that GSH deficiency plays a major role in augmenting SARS-CoV-2 oxidative damage, which leads to greater disease progression and mortality. This viewpoint was based on data showing lower GSH and higher ROS levels in COVID-19 patients with mild disease and increasing severity that included higher viral load with GSH deficiency [166] and work completed by Hurwitz et al. [167] that demonstrated improvement in dyspnea with high dose oral and IV GSH in two patients with underlying conditions who tested positive for COVID-19. These conclusions were based on very small samples and therefore require additional larger clinical studies to replicate the findings and eventual intervention studies. The evidence presented above suggests that Se availability contributes to resisting SARS-CoV-2 infection, corresponding with studies that show adequate levels of Se status maintains an appropriate immune response to viral infection [6,134,136]. 

There are no known published Se supplementation clinical trials in the context of COVID-19 at this time and one study is currently listed on clincaltrials.gov that will examine the efficacy of Se (selenious acid infusion also known as sodium selenite) for the treatment of moderately-ill, severely ill, and critically ill COVID-19 patients (Identifier: NCT04869579). Sodium selenite supplementation has been proposed for the prevention of COVID-19 infections and severe disease [149,168]. Sodium selenite is easily available, short-term toxicity is marginal and may cross the blood-brain barrier [149]. This chemical form may oxidize thiol groups located in the virus protein disulfide isomerase, which would interfere with its ability to infiltrate the cell membrane and produce an infection [168]. TXNRD activity increases quickly after supplementation with sodium selenite in cancer cell lines and critically ill patients [169,170] and has demonstrated reduced ROS production and viral-induced cell apoptosis in cell culture studies [171]. A common feature of COVID-19 is thrombotic complications and altered platelet function is believed to affect the sequelae of this infection [172]. Sodium selenite has also been shown to have an anti-aggregating effect through its reduction of thromboxane A2 formation, an important factor in blood platelet activation and formation [173]. The effectiveness of sodium selenite for the prevention and management of COVID-19 should be tested immediately as the COVID-19 pandemic continues to persist and threaten the health of individuals globally, thus necessitating rapidly accessible treatment strategies. 

Since Se has pronounced therapeutic potential for the treatment of viral infections and other conditions such as cancer, Se nanomedicine has received a lot of attention. Se nanoparticles are known to have low toxicity with marked and selective cytotoxic effects with small quantities [174]. Additionally, Se nanoparticles have high effectiveness in the inhibition of oxidative damage [175,176,177]. Recently published data show that Se nanoparticles activate programmed cell death in target cancer tissue through calcium (Ca)^2+^ signaling pathways [178]. Immune cells also require calcium flux to generate oxidative stress [174]. Through chemical methods, Se nanoparticles may be produced with Se sources that include sodium selenite, selenious acid, and sodium selenosulfate [174]. Due to the developing relationship between Se and COVID-19, Se nanomedicine is being suggested as a tool in the fight against SARS-CoV-2 [179]. Currently, there are tremendous prospects of using nanomedicine in ARDS for the prevention, diagnosis, and treatment, which may have applicability for COVID-19 [180]. Jin et al. [181] discovered that an organic Se compound known as Ebselen, and a promising antioxidant drug, could inhibit SARS-CoV-2 by penetrating the cell membrane and displaying antiviral activity. Ebselen is known to have anti-inflammatory activity, mimic GPX activity, and should be considered for clinical studies [181,182]. 

## 5. Nutrition and Recommended Intakes and Supplementation of Selenium

Optimal nutrition is important for regulating inflammatory and oxidative stress processes within the body [183]. These processes are important for the maintenance of the immune system and previous research has shown that nutritional status affects health outcomes in viral infections [13]. The number of chronic conditions globally has increased [184] and seems to have a strong influence on the disease progression of COVID-19 [185]. Therefore, a healthy dietary pattern, a modifiable risk factor, may reduce chronic conditions, the development of infections, and the severity of viral infections [186,187]. Low intakes of Se, zinc, magnesium, copper, vitamins A, B6, B12, C, D, and E, and omega-3 fatty acids have been associated with worse outcomes in viral infection and lower immunity [188,189,190] and should also be considered for the prevention and management of COVID-19 [191,192,193]. More research is needed to further define the role of nutrition in COVID-19 infection and disease progression and appropriate doses. However, at a minimum in order to support the functions of the immune system, recommendations for the consumption of nutrients that may impact immunity should be the in amounts directed by the reference nutrient intakes or recommended daily allowance [187]. 

Intake of Se by humans may vary according to differences in sources of food, accumulation of Se in animals and the content of Se in the soil [8]. Countries with poor Se in the soil include Finland, New Zealand, the United Kingdom, sub-Saharan Africa, and certain areas of China where Keshan is prevalent [194,195]. Consequently, the differences in intake of Se may be quite large, for example, daily Se intake in Europe is estimated to be about 40 µg per day and in the U.S.A. about 90–134 µg per day [196]. Selenium is plentiful in Brazil nuts, seafood, organ meats, muscle meats, cereals, grains, and dairy [197], and the diet in the U.S.A. provides Se mainly from grains, meat, poultry, fish, and eggs [198]. Selenium in vegetables is predominantly found as selenomethionine, selenium-methylselenocysteine or γ-glutamyl-selenium-methylselenocysteine and in meat as selenocysteine [8]. Inorganic Sec compounds including sodium selenite and selenate may be found in dietary supplements [8].

The current recommended intake for Se in the U.S.A. for adults is 55 µg per day. This recommendation is based on the consumption of Se needed to maximize the action of the selenoprotein GPX [199]. The WHO recommended nutrient intakes for Se in adults are 26 µg per day for women and 34 µg per day for men [200]. The Tolerable Upper Intake Level (UL) for Se in adults is 400 µg per day and the limit is based on the increased risk for selenosis [199]. The European Food Safety Authority in the European Union set the daily adequate intake for Se at 70 µg [201]. Selenosis in humans may cause loss of hair, thickened and stratified nails, and a garlic-like odor present in the mouth and skin [202,203]. Toxicity of Se appears to be less common than Se deficiency and has been reported to be caused by over supplementation and accidental consumption of high doses through consumption of foods grown in soil with large amounts of Se present [9,204]. 

## 6. Conclusions

Selenium plays an important role in the host during viral infections, assisting in redox homeostasis, antioxidant defense, and minimizing oxidative stress (Table 1). These protective roles are accomplished largely through its incorporation into selenoproteins. Antioxidant defense systems that incorporate selenoproteins, mainly GPXs and TXNRDs, are crucial for reducing oxidative stress created by an imbalance of ROS as a result of viral infections. Selenium deficiency may also have an effect on the viral genome leading to greater pathogenicity. Adequate Se intake is imperative for these systems to be functional and provide full enzymatic activities. The data on the relationship between Se and the novel SARS-CoV-2 are still evolving, however, preliminary results show a link between Se status and severity of COVID-19 outcomes. Therefore, Se status should be reviewed in patients with COVID-19 as a risk factor for graver outcomes. The literature on RNA viruses provides promising mechanisms of action for the use of Se in the prevention and disease management of COVID-19. Sodium selenite has been proposed as a preventive measure and adjuvant therapy for COVID-19 based on its potential ability to restore GPX and TXNRD activity, reduce viral-induced cell apoptosis, protect endothelial cells, and reduce blood platelet aggregation. Se nanoparticles should also be considered as a mechanism to deliver Se to target organs such as the lungs and deliver Se without risks of toxicity. Data available from other viral infections in conjunction with the current COVID-19 data provide sufficient justification for future and timely Se intervention studies. 

## Figures and Tables

**Table 1:** Summary of Selenium Studies.

Topic	Conclusions	References
Viral Infections, Reactive Oxygen Species (ROS), and Selenium (Se)	Viral Infections are associated with ROS. Glutathione peroxidases (GPXs) and thioredoxin reductases (TXNRDs) (family of selenoproteins) play a role as antioxidants and confer protection against free radicals as a result of viral infection. Se intake may affect GPXs and TXNRDs levels.	[8,35,36,40,42]
Coxsackie Virus	Keshan disease responsive to sodium selenite supplementation. Keshan disease due to infection with Coxsackie B virus and Se deficiency. Benign Coxsackie B virus became virulent when mice were Se-deficient and greater pathology in cardiovirulent Coxsackie B virus strain. Se deficiency was responsible for a change in the genotype of the benign coxsackie virus CVB3/0 that caused it to become virulent and decreased the activity of GPX.	[28,45,47,48,49,50,51,52,54]
Influenza	Se deficiency has been associated with poor selenoprotein expression, altered antioxidant response, and viral genome changes in viral influenza A infection. Se supplementation in healthy older adults yielded beneficial and detrimental effects related to anti-flu immunity.	[11,12,13,55,56]
Human Immunodeficiency Virus (HIV)	Se deficiency was associated with advanced immunodeficiency and mortality. Se supplementation in HIV has demonstrated benefits on HIV disease progression.	[63,68,69,70,71,86,87,88,91,92,93,94,95,96]
Hepatitis B and C Viruses	Se levels associated with HBV/HCV infection, severity, and progression of disease. Depletion of GSH and GPX in HBV/HCV. Se supplementation in areas of low intake may prevent HBV and primary liver cancer. Se deficiency associated with inflammation of the liver.	[103,104,105,106,117,118,125,129]
Poliovirus	Supplementation of Se to improve the response of polio vaccine remains inconclusive.	[135,136]
Severe Acute Respiratory Syndrome Coronavirus 2 (SARS-CoV-2)	Se soil status may be associated with COVID-19 incidence and severity of COVID-19 outcomes in China. COVID-19 infection and severity associated with lower Se levels, greater oxidative stress, and lower antioxidant status.	[158,159,161,162,163,164,165,166,167]

## Data Availability

Not Applicable.
